# Experiences from COVID-19-driven use of telephone consultations in a cardiology clinic—The CoviTel study

**DOI:** 10.1371/journal.pone.0273492

**Published:** 2022-10-19

**Authors:** Astrid Brink Hundebøll, Stine Rosenstrøm, Magnus Thorsten Jensen, Ulrik Dixen

**Affiliations:** 1 Department of Cardiology, University of Copenhagen, Amager Hvidovre Hospital, Copenhagen, Denmark; 2 Department of Public Health, Section for Nursing, University of Aarhus, Aarhus, Denmark; 3 Centre for Advanced Cardiovascular Imaging, Queen Mary University of London, London, United Kingdom; 4 Faculty of Health and Medical Sciences, Department of Clinical Medicine, University of Copenhagen, Copenhagen, Denmark; PLOS ONE, UNITED KINGDOM

## Abstract

**Introduction:**

The COVID-19 pandemic triggered a rapid shift towards telephone consultations (TC) in the out-patient clinic setting with little knowledge of the consequences. The aims of this study were to evaluate patient-centred experiences with TC, to describe patterns in clinical outcomes from TC and to pinpoint benefits and drawbacks associated with this type of consultations.

**Methods:**

This mixed methods study combined an analysis of quantitative and qualitative data. A quantitative, retrospective observational study was conducted employing data from all 248 patients who received TC at an out-patient cardiology clinic during April 2020 with a one-month follow-up. Semi-structured interviews were conducted; Ten eligible patients were recruited from the outpatient clinic by *purposive sampling*.

**Results:**

Within the follow-up period, no patients died or were acutely hospitalised. Approximately one in every four patients was transferred to their general practitioner, while the remaining three-quarter of the patients had a new examination or a new consultation planned. The cardiologist failed to establish contact with more than a fifth of the patients, often due to missing phone numbers. Ten patients were interviewed. Five themes emerged from the interviews: 1) *Knowing an estimated time of the consultation is essential for patient satisfaction*, *2) TC are well perceived when individually adapted*, *3) TC can be a barrier to patient questions*, *4) Video consultations should only be offered to patients who request it*, and *5) Prescriptions or instructions made via TC do not cause uncertainty in patients*.

**Conclusions:**

The TC program was overall safe and the patients felt comfortable. Crucial issues include precise time planning, the patient’s availability on the phone and a correct phone number. Patients stressed that TC are unsuitable when addressing sensitive topics. A proposed visitation tool is presented.

## Introduction/Background

During the global COVID-19 pandemic [1], life patterns have undergone dramatic changes. In health care, COVID-19 has accelerated the use of telemedicine or telephone consultations (TC) to facilitate social distancing while upholding healthcare services [2].

On 11 March 2020, all Hospitals in Denmark were instructed by the Danish Government to prepare an almost total lockdown on scheduled visits to minimize the spread of COVID-19. Thus, within a few days after 11 March, the out-patient clinic at the Department of Cardiology, University of Copenhagen, Amager Hvidovre Hospital, Denmark, had cancelled all non-acute visits and from 23 March TC was planned to replace face-to-face visits.

Previously, TC had only rarely been used, as face-to-face consultation had been the default consultation method at the outpatient clinic. The rapid COVID-19-driven shift towards TC consultations replacing face-to-face consultations was challenging as local experience with TC consultations was limited.

Before the COVID-19 pandemic, studies had established the overall safety of the use of TC. Even so, previously TC was mainly used on a smaller scale and often limited to a few diagnose-specific fragile patients with chronic diseases such as chronic obstructive pulmonary disease and heart failure [3,4].

The future course of the COVID-19 pandemic remains unknown. However, as part of the general precautions in both society and within healthcare, a more widespread change towards using virtual consultations has emerged worldwide, and studies have shown sufficient patient satisfaction with telehealth owing to its convenience and a reduced risk of infection [5,6].

The primary aim of this study was to evaluate patient-centred experiences with TC. Secondly, we aimed to describe patterns in clinical outcomes in TC such as frequency of referrals back to primary healthcare and successful telephone contact. Finally, we aimed to pinpoint the benefits and drawbacks associated with TS consultations and to present a guide for future TC visitation.

## Methods

### Overall study design and population

The study was conducted as a mixed-methods study offering a combined analysis of quantitative and qualitative data [7,8]. We employed a sequential technique gathering in which the two types of data were gathered independently and subsequently analysed to achieve a comprehensive interpretation of the research focus [7].

A quantitative, retrospective, observational study was conducted including all patients who were rescheduled during April 2020 to a TC instead of receiving an in-person consultation at the outpatient clinic of the Department of Cardiology, University of Copenhagen, Amager Hvidovre Hospital, Denmark. The quantitative data were supplemented by individual in-depth interviews with ten patients to uncover patient-perceived TC quality.

### Study setting

The study was conducted at the out-patient clinic of the Department of Cardiology, University of Copenhagen, Amager Hvidovre Hospital, Denmark. Amager Hvidovre Hospital is one of six hospitals with a Department of Cardiology located in the Capital Region of Denmark. Approximately 40,000 annual patients are treated in the outpatient clinic, which makes it one of the largest Danish outpatient clinics. In Denmark, all residents are covered by publicly financed healthcare, which ensures free and equal healthcare access. The hospital service area of the Department of Cardiology, University of Copenhagen, Amager Hvidovre Hospital is, therefore zip code-based. The most common cardiac diseases treated are ischaemic heart disease, heart failure, arrhythmias, heart valve diseases and aortic diseases.

All authors were employed at the study site at the time of this study. We felt obliged to investigate the outcomes of this sudden change of consultation method by conducting this exploratory study to gain awareness of TC benefits and drawbacks and to allow patients to voice their experiences.

Before the Danish government initiated the national lockdown in March 2020, in-person consultations were the default modality used for outpatient visits. Immediately after the lockdown was announced, most physical attendance consultations were replaced by a COVID-19-driven telephone program. The staff were senior cardiologists with no previous formal training in TC. The patients receiving the COVID-19-driven telephone program were stable, attending a clinical out-patient follow-up course and did not need imaging examinations such as echocardiography for which physical attendance is required. All clinical decisions and plans for follow-up were documented as usual in the electronic health records. The patients were given relevant contact data allowing them to reach out to the out-patient clinic if they needed further information following their TC.

### Data extraction–Quantitative analysis

A total of 248 patients had TC consultations during April 2020. A one-month follow-up was registered comprising registration of hospitalization, death, and additional telephone contact to the out-patient clinic. All patient files were screened; and patient data regarding age, gender, primary cardiac diagnosis and type of scheduled consultation were registered. Moreover, the practical/logistical outcome of the TC was registered: no contact made, new examination planned, new consultation planned, missed planned examinations, or referral back to the general practitioner. The outcome “Missed planned examinations” describes the situation in which the cardiologist failed to order the planned examination correctly due to new routines in the TC setting. Investigator ABH conducted the quantitative data analysis.

### Interviews–Qualitative data

Semi-structured interviews were conducted at the Department of Cardiology, University of Copenhagen, Amager Hvidovre Hospital, Denmark. Interviews aimed at uncovering patients’ experiences with TC. A predetermined interview guide was developed by the authors to guide semi-structured interviews. This approach was chosen to achieve as many thoughts and viewpoints from the patients as possible [9]. All interviews were conducted by the first (ABH) or the second author (SR), both of whom have experience conducting qualitative research interviews. The first interview was conducted as a pilot interview to assess the questions of the interview guide and avoid any misleading, irrelevant and unclear wording. ABH conducted the pilot interview and SR was present during the interview. No changes were made to the interview guide following the pilot interview. The interview guide is displayed in full in Table 1.

**Table 1:** Semi-structured interview guide.

Research questions	Interview questions
How did the patient experience the telephone conversation?	Did you and the doctor talk via mobile phone or landline?
	Where were you during the conversation?
	Did you have any relatives participating in the conversation via speakerphone?
	Did you know the time of the incoming call?
	What significance did it have to you that you knew/did not know the time of the call?
	Do you think that the issue regarding your health discussed over the telephone was suitable to discuss during a telephone consultation?
Did the patient experience any certainty or uncertainty during the telephone consultation?	Did any specific aspects of the conversation make you feel certain or uncertain?
	Have you felt or did you feel any certainty or uncertainty regarding the changes made in your medication?
	Have you felt or did you feel any certainty or uncertainty regarding the planning of new medical consultation?
How did the patient experience the communication with the doctor during the telephone consultation?	Did the doctor introduce him/herself to you?
	Did you know the doctor from previous consultations?
	Did the doctor ask about your civil registration number or did he/she in any other way confirm your identity?
	How did you find the information the doctor gave you?
	Did you find it thorough enough?
	Did you find you had an option to ask questions?
Would the patient prefer video consultations?	Did it affect the conversation that you could not see the doctor?
	Would you prefer if the doctor had called via video link?
	Would it be possible for you to accept a call via video link?
Patient’s perception and future preferences?	What do you think could be the potential advantages or disadvantages of telephone consultations?
	Would you prefer a telephone consultation in the future?
	Is there anything you would like to share or on which you want to elaborate?

Eligible patients were recruited from the outpatient clinic for the COVID-19-driven telephone program using a *purposive sampling strategy* [10]. Thus, we aimed to include patients who differed in terms of age, gender, primary cardiac diagnosis and number of previous cardiac consultations to achieve rich data. To establish if complex information was understood during TC, we added as an inclusion criterion that patients had to have had a change in medication as part of their consultation. Patient characteristics are presented in Table 4.

Patients were contacted by telephone on the day of their TC and invited to participate in an interview the following day. Subsequently, written information was sent to them by secure email. Oral informed consent was given by the patient at the beginning of each interview. The interviews were conducted as telephone calls which were digitally recorded and transcribed verbatim in an anonymized form by two secretaries. Interviews were conducted until *data saturation* had been reached; data saturation is the point at which new interviews generate no further aspects or insights enriching our understanding of patients’ experience with TC [11].

Subsequently, the interviews were analysed using qualitative content analysis [12]. In this analysis method, the written interviews were read repeatedly and then coded focusing on their contents and the underlying meaning. *Condensation* describes the process of shortening the contents while preserving its core meaning. Several *sub-themes* were condensed from the analysis process and were then grouped into *themes*.

Evaluation of the credibility, dependability, confirmability and transferability [12,13] was done when designing the study. Study credibility was strengthened through the qualitative content analysis, which was conducted by the investigators ABH, SR and UD each of whom verified the interpretations individually. Subsequently, all discrepancies were discussed until an agreement was reached.

To avoid confirmability, we sought to acquire knowledge by reading scientific literature. Thereby, we did not rely exclusively on *a priori* knowledge and our preconceptions when developing the interview guide and analysing the data. To ensure the dependability of the study, the research team was transparent about how the data collection and interpretation were conducted [14]. The study findings contribute general new knowledge. Transferability to other contexts is best assessed by others [15].

### Statistics

Descriptive analysis was performed using the Excel program.

### Ethics

The study conforms to the principles outlined in the Declaration of Helsinki [16]). All interviewed participants received written and oral information about the study and gave informed consent. Data were handled, processed and analysed maintaining confidentiality, and then anonymized. The Danish Data Protection Agency and the Local Ethical Committee, Capital Region of Denmark, was approached, and the study followed the National Ethical Guidelines (no. 11052) [17].

## Results

### Quantitative results

In the quantitative part of the study, 248 patients were registered. Patient characteristics are described in Table 2. None of the patients was lost to follow-up. Within the one-month follow-up period, no patients were admitted acutely to the hospital or died. Slightly more than one-fourth of the patients were referred back to their general practitioner (27.8%). Approximately half of the patients (50.4%) had new examinations planned (a subsequent consultation was scheduled). 15.3% had a scheduled subsequent consultation with a doctor and 5.6% with a nurse. Two of the 248 patients (0.8%) had “missed planned examinations”. In 2.4% of the cases, patients subsequently contacted the staff (nurses mainly) with follow-up questions to the TC. Follow-up data are presented in Table 3.

**Table 2:** Patient characteristics, quantitative study.

**Patient characteristics**				
	Number of patients	248		
	Age, mean		61.1 years	(17–90)
	Gender	Female	117	(47.1%)
		Male	131	(52.8%)
**Patient characteristics in primary diagnosis groups**				
*Ischaemic heart disease*				
	Number of patients	70	28.2%	
	Age, mean		63.7 years	(39–83)
	Gender	Female	27	(38.6%)
		Male	43	(61.4%)
	Was TC first contact?	Yes	4	(5.7%)
		No	66	(94.3%)
*Arrhythmia*				
	Number of patients	103	41.5%	
	Age, mean		59.5 years	(18–90)
	Gender	Female	45	(43.7%)
		Male	58	(56.3%)
	Was TC first contact?	Yes	18	(17.5%)
		No	85	(82.5%)
*Thromboembolic disease*				
	Number of patients	23	9.3%	
	Age, mean		60 years	(33–85)
	Gender	Female	15	(65.2%)
		Male	8	(34.8%)
	Was TC first contact?	Yes	0	(0.0%)
		No	23	(100%)
*Heart valve disease*				
	Number of patients	14	5.6%	
	Age, mean		74.1 years	(44–86)
	Gender	Female	8	(57.1%)
		Male	6	(42.9%)
	Was TC first contact?	Yes	2	(14.2%)
		No	12	(85.8%)
*Other diagnoses including fast-track cardiac examinations*				
	Number of patients	38	15.3%	
	Age, mean		56,1 years	(17–77)
	Gender	Female	22	(57.9%)
		Male	16	(42.1%)
	Was TC first contact?	Yes	7	(18.4%)
		No	31	(81.6%)

**Table 3:** Results, quantitative study.

Results, quantitative study			
	Total number of patients scheduled for TC, April 2020	248	
*Was TC first contact*?			
	TC in April 2020 was the first contact	31	12.5%
	Previous contact before TC in April 2020	217	87.5%
*Was contact established*?			
	No contact made	39	15.7%
	Contact made to someone else (wife/ child /relative)	16	6.5%
	Contact established with patient	193	77.8%
*Outcome for TC April 2020*			
	Discharged, referred back to GP	69	27.8%
	New examination and consultation with doctor scheduled	125	50.4%
	New consultation with doctor scheduled	38	15.3%
	New consultation with nurse scheduled	14	5.6%
	“Missed planned examinations”	2	0.8%
	The patient was admitted to the hospital due to TC	0	0.0%
*30-day follow-up after TC in April 2020*			
	2nd attempt of TC with patients where no contact had been made during the first scheduled TC	26	10.5%
	The patient contacted the department with questions after TC	6	2.4%
	The patient was admitted to the hospital; cardiac cause	3	1.2%
	The patient was admitted to the hospital; non-cardiac cause	15	6.0%
	Discharged patient was re-referred under the same cardiac diagnosis	0	0.0%
	Patient died, assumed cardiac cause	0	0.0%
	Patient died, assumed non-cardiac cause	0	0.0%
	No unscheduled contact was provided during the 30-day follow-up	198	79.8%

### Interview results

Ten patients (5 M, 5 F) with an age span from 22 to 80 years were interviewed; all had a minimum of one cardiac disorder and all had changes introduced to their medication during their TC. Patient characteristics are presented in Table 4. No one declined the invitation to participate. No repeat interviews were made. The mean length of the interview was 17.9 minutes (range 12 minutes; 52 seconds– 26 minutes; 46 seconds).

**Table 4:** Patient characteristics, qualitative interviews.

No.	Age *Years*	Sex *M/F*	Primary cardiac diagnosis ICD-10-CM	Change in medication *Inclusion criteria*	Researcher initials
1	79	F	I49.3 Ventricular premature depolarization	Yes	ABH
2	37	M	D47.3 Essential thrombocythemia	Yes	ABH
3	60	M	I71.9 Aortic aneurysm UNS without rupture	Yes	SR
4	77	F	I35.0 Non-rheumatic aortic (valve) stenosis	Yes	SR
5	59	M	I35.1 Non-rheumatic aortic (valve) insufficiency	Yes	SR
6	72	F	I50.9 Heart failure, unspecified	Yes	SR
7	61	F	DZ035A Unknown cause of chest pain	Yes	ABH
8	64	M	I48.0 Paroxysmal atrial fibrillation	Yes	SR
9	55	M	I49.3 Ventricular premature depolarization	Yes	ABH
10	22	F	DZ035A Unknown cause of chest pain	Yes	ABH

The patient-centred data from the interviews were analysed and the following five themes emerged.

### Knowing the exact time of the consultation is essential for patient satisfaction

The patients reported that not knowing the precise time of the scheduled call made them feel like their whole day was tied up. The patients planned their day around the expected call, and they wished to be in a suitable private setting when receiving the call. They reported feeling overlooked when their call came through much later than expected.

### Telephone consultations are well perceived when individually adapted

In general, patients had a positive experience with the TC. Many emphasized shorter transport time and less time waiting. One patient with parallel hospital courses reported a greater sense of freedom when consulted over the phone. All patients reported that messages categorized as *tough* or *sensitive* should not be given by telephone, largely due to the difficulty associated with providing psychological support. In line herewith, we observed a pattern that milder messages, i.e. normal test results or benign arrhythmias, were suitable for TC.

### Telephone consultations may be a barrier to patient questions

Many patients had taken time to prepare questions before their consultation and felt that they were allowed to ask their questions. Nevertheless, many patients reported that TC may be a barrier to asking their questions, especially because it is impossible to ask “last minute” questions after the call had been concluded, as opposed to asking a “Doorknob question”. Some patients requested having support from relatives and expressed concerns about this aspect of TC. This finding is illustrated in Table 5.

**Table 5:** Examples from the qualitative content analysis; from code to theme.

Code	Sub-theme	Theme	Sample Quote
The patients contemplate that clinical consultations make it more likely for them to ask questions than telephone consultations.	Clinical consultations can be a facilitator for the patients to ask questions	Telephone consultations may be a barrier for patients to ask questions	*“If I had been in the room with him* [the doctor], *then I could have asked supplementary questions as they popped into my mind afterwards*. *When the phone is hung up*, *you just can’t do that*. *“Oh*, *I forgot to ask about that*.*”—*Patient 5
“Doorknob questions” are more difficult to ask over the phone			
More difficult to include relatives	Clinical consultations make it easier to include relatives		*“My girlfriend is a bit unhappy that she didn’t take part… She would have liked to participate so she could have listened*, *observed and asked questions as well”—*Patient 3
More difficult for relatives to ask questions			
The patients wish to have support from relatives when having a more serious conversation.			
The patients may feel unsure about what is expected of them before a telephone consultation	It is important to align expectations before a telephone consultation		*“It’s more difficult when it’s on the telephone*. *It changes the context; I didn’t know how long time it was going to take or what we were going to talk about… I didn’t know who was going to call me*. *It could have been anyone; a doctor or a nurse”–*Patient 7
Telephone consultations are more suitable when the patient knows what the conversation is about			
Patients wish to engage actively in the consultation			
Telephone consultations require that the patient takes a different approach to preparation than a clinical consultation	It may be a barrier for questions if the doctor calls at a time not known in advance		*“I wasn’t prepared when the doctor called*. *I wasn’t aware he would call at that moment*. *It wasn’t till afterwards that I thought to myself*: *“Oh*, *I should’ve asked*.* *.* *.* *.*”* *-* Patient 9
It is important for patients to mentally prepare themselves before a consultation with a doctor			
Mental preparation is difficult when the patient does not know the time of the call			

### Video consultations should be offered only to patients who request it

When asked to reflect upon the possibilities of video consultation, the patients reported that video may be relevant in some circumstances, and several patients mentioned showing a rash to their general practitioner as an example hereof. A few patients had previously had video consultations with their general practitioner and did not feel that this would outperform TC in terms of adding a personal aspect to the conversation.

It is, however, important to acknowledge that the patients did consider video consultation to be potentially beneficial under some circumstances–just not to see the doctor’s face.

### Prescriptions or instructions made by telephone consultations do not cause uncertainty in patients

The research interviews thoroughly investigated if the patients had experienced any uncertainty regarding the changes made in their medication, planning of new appointments or further examinations. No patients reported a feeling of uncertainty, and all felt that they had been thoroughly informed by their doctor.

## Discussion

In this mixed-methods study of operational outcome and patient-centred evaluation of TC during the COVID-19 crisis, we found that using TC was an overall safe and well-accepted strategy, but several crucial points needed to be addressed.

### Quantitative data

With the limitation of a short follow-up period, the quantitative data showed no risk of acute hospitalization or death. In addition, only very few patients had “missed planned examinations” (0.8%) or needed additional contact with the outpatient clinic (2.4%).

However, the cardiologist failed to establish contact with more than a fifth of the patients as no contact was made in 15.7% of the cases; moreover, in 6.5% of the cases, contact could only be established with a relative of the patient. Studying the file notes, we observed that in most cases, failure to reach the patient was due to simple explanations such as missing information about contact telephone numbers in the electronic file. Simple preparation standards such as mandatory registration of telephone numbers may serve to eliminate these obstacles. The setting of this study did not allow for evaluation of the quality of alternative contacts, i.e. cases where the doctor did not reach the patient but instead spoke to a relative or a healthcare worker (6.5% of all cases).

Of interest, only one in four patients were referred back to their general practitioner as a result of the TC (27.8%), whereas the remaining part of the patients was referred to either new examinations plus an additional consultation (50.4%) or a consultation with a doctor (15.3%) or a consultation with a nurse(5.6%). Although difficult to assess in this study population, this may reflect a barrier against concluding an outpatient clinic course during a TC.

### Interview data

Several important themes emerged from the interviews in the patient-centred part of the study. In general, patients felt secure and comfortable with TC.

However, the patients needed to have an exact appointment time; this was an important point for all participating patients. The patients felt tied up all day if the TC was postponed without advance notice, and they frequently felt “caught” during other activities when the TC finally came through. This inconvenience can easily be solved by booking the patient into TC schedules with prespecified time slots.

Also, all patients clearly expressed a need for individualization where the nature of the health problem of the consultation was taken into account in the decision to do a TC or face-to-face consultation. Specifically, the patients distinguished between consultations in which a **“**good message**”** or benign results were given as opposed to receiving complex information or having to discuss a serious cardiac condition. In the latter situation, all patients would prefer a face-to-face consultation with the doctor and having the option to bring a relative with them. Accordingly, important principles such as patient involvement, family activation in care and shared decision-making in the TC setting require specific attention [18–20].

Surprisingly, only one patient, who himself worked with ICT, expressed a wish for optional video consultations. All other patients preferred TC or face-to-face consultations. An American study by Lion et al. discussed the challenges of digital literacy, internet access and language skills in the shift towards virtual consultations [21]. The authors discussed the use of TC versus video consultations and noted that only video consultations allow non-verbal communication with gestures and reading of body language. The authors note this as an area of particular relevance in fragile patients and communities with difficult socio-economic conditions.

We had chosen change in medication as an inclusion criterion to evaluate if complex information was understood via TC. Our study reassuringly found that none of the interviewed patients felt unsure about changes in medication or instructions given over the telephone. This is an important aspect to investigate in future studies to continuously assess patient safety.

**Fig 1:**
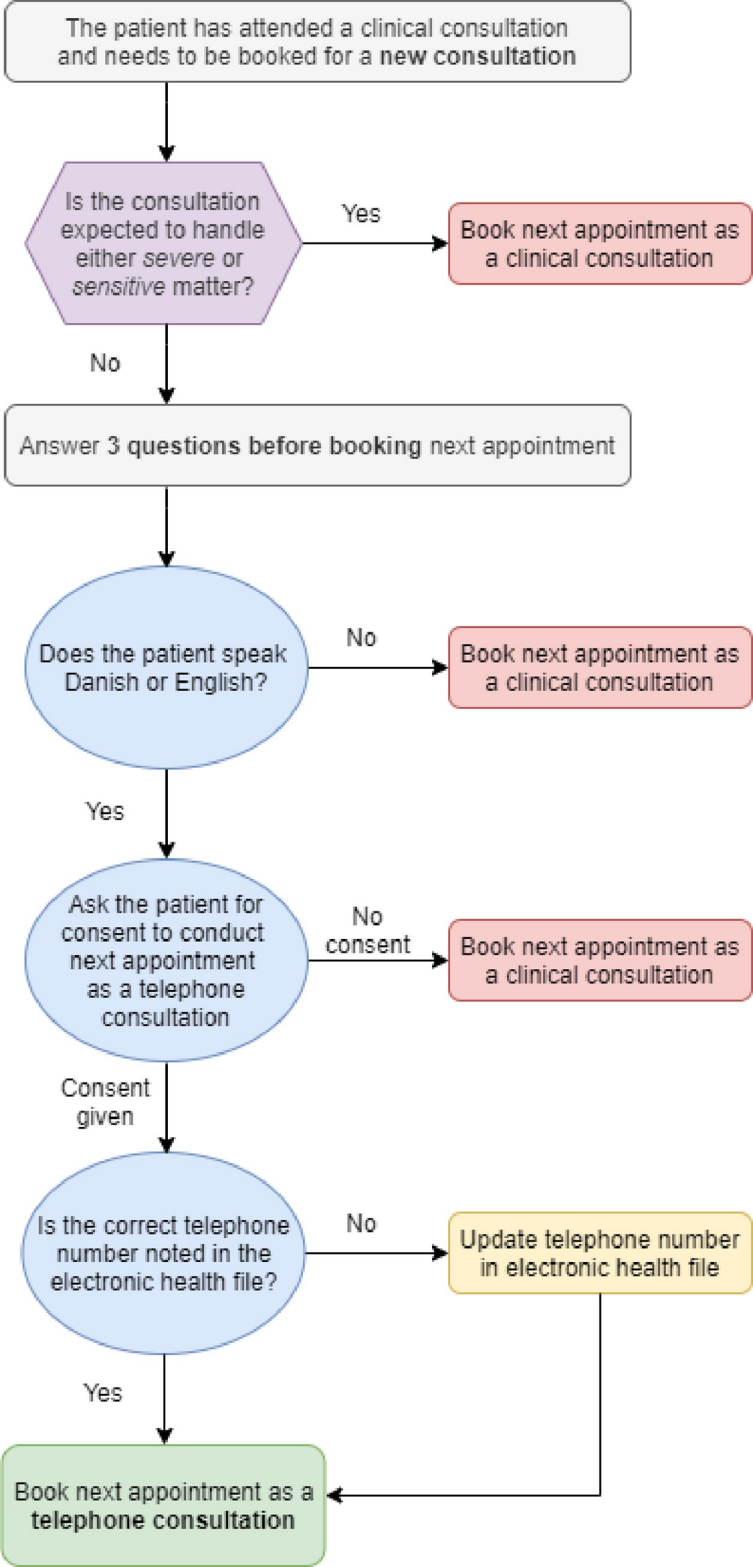
Proposed visitation tool for booking telephone consultations and clinical consultations with patients.

As TC will most likely become a necessary element in outpatient clinic work methods in the future, we propose a short guidance tool that may be used for TC visitation and that may be adjusted locally. In Fig 1, we listed the main key notes that the staff should be aware of when introducing new consultations patterns. We believe that our visitation tool may be used in the broader context of telehealth or telemedicine, e.g. for video consultations (VC). Research focusing on VC has found results similar to ours; general patient satisfaction, overall safety and a patient-reported preference for VC when the health concern addressed was of a familiar or routine nature [6–22].

### Limitations

The qualitative results were limited to views from patients and did not include, e.g., doctor or nurse views. When using purposive sampling, a risk of *selection bias* exists. Of notice, qualitative research seeks an exploratory understanding of a phenomenon, not a causal explanation. Limitations to this methodology involve that findings may not be directly transferable to other settings. The quantitative results were affected by the acute circumstances of the shift to TC and, therefore, do not mirror standard out-patient clinic practice.

## Conclusions

This mixed-methods study found that the patients who consulted via TC due to the COVID-19 pandemic had no risk of acute hospitalization, and no deaths occurred within the follow-up period. Very few patients needed extra contact to the out-patient clinic (2.4%).

Approximately one in every four patients was referred back to their general practitioner (27.8%), while the remaining three-quarters of the patients had a new examination or a new consultation planned. The cardiologist failed to establish contact to more than one fifth of the patients as no contact was made in 15.7% of the cases; and in 6.5% of the cases, contact could be established only to a relative of the patient. In cases in which contact was not made, this was typically due to simple issues, e.g., no or an incorrect phone number. Overall, patients felt comfortable with the TC. However, when deciding between a face-to-face consultation or a TC, several issues need to be addressed; most importantly, the complexity and sensitivity of the health concern explored. All patients reported a preference for face-to-face consultation when the health concern addressed was of a sensitive nature. Other crucial issues included language skills, a need for precise time planning and the patient’s availability on the phone. Finally, this study proposed a visitation tool for patient TC booking based on the findings of the study. Using this visitation tool may potentially serve to increase patient satisfaction.

## Supporting information

S1 Dataset (XLSX)
